# Double Roles of Macrophages in Human Neuroimmune Diseases and Their Animal Models

**DOI:** 10.1155/2016/8489251

**Published:** 2016-03-13

**Authors:** Xueli Fan, Hongliang Zhang, Yun Cheng, Xinmei Jiang, Jie Zhu, Tao Jin

**Affiliations:** ^1^Department of Neurology and Neuroscience Center, First Hospital of Jilin University, Jilin University, Changchun 130021, China; ^2^Department of Neurobiology, Care Sciences and Society, Karolinska Institute, 141 86 Stockholm, Sweden

## Abstract

Macrophages are important immune cells of the innate immune system that are involved in organ-specific homeostasis and contribute to both pathology and resolution of diseases including infections, cancer, obesity, atherosclerosis, and autoimmune disorders. Multiple lines of evidence point to macrophages as a remarkably heterogeneous cell type. Different phenotypes of macrophages exert either proinflammatory or anti-inflammatory roles depending on the cytokines and other mediators that they are exposed to in the local microenvironment. Proinflammatory macrophages secrete detrimental molecules to induce disease development, while anti-inflammatory macrophages produce beneficial mediators to promote disease recovery. The conversion of the phenotypes of macrophages can regulate the initiation, development, and recovery of autoimmune diseases. Human neuroimmune diseases majorly include multiple sclerosis (MS), neuromyelitis optica (NMO), myasthenia gravis (MG), and Guillain-Barré syndrome (GBS) and macrophages contribute to the pathogenesis of these neuroimmune diseases. In this review, we summarize the double roles of macrophage in neuroimmune diseases and their animal models to further explore the mechanisms of macrophages involved in the pathogenesis of these disorders, which may provide a potential therapeutic approach for these disorders in the future.

## 1. Introduction

Macrophages distributed in tissues throughout the body play a key role in immune response, tissue homeostasis, metabolism, and repair [1]. Mature macrophages in different tissues present with different phenotypes, such as microglia in the brain, alveolar macrophages in the lungs, Kupffer cells in the liver, and osteoclasts in bone tissue [2]. In addition, macrophages can switch their phenotypic and functional properties depending on the signals in their microenvironment in homeostasis and disease [3]. The polarization of macrophages is determined by the cytokines and other mediators they encounter. Different subsets of macrophages exert either proinflammatory or anti-inflammatory roles. Recently, the studies have demonstrated that macrophages take part in the pathological process of neuroimmune diseases. This review outlines the double roles of macrophages in human neuroimmune diseases, such as multiple sclerosis (MS), neuromyelitis optica (NMO), myasthenia gravis (MG), and Guillain-Barré syndrome (GBS) as well as their animal models.

## 2. An Overview of Macrophages

### 2.1. The Origin of Macrophages

**Figure 1:**
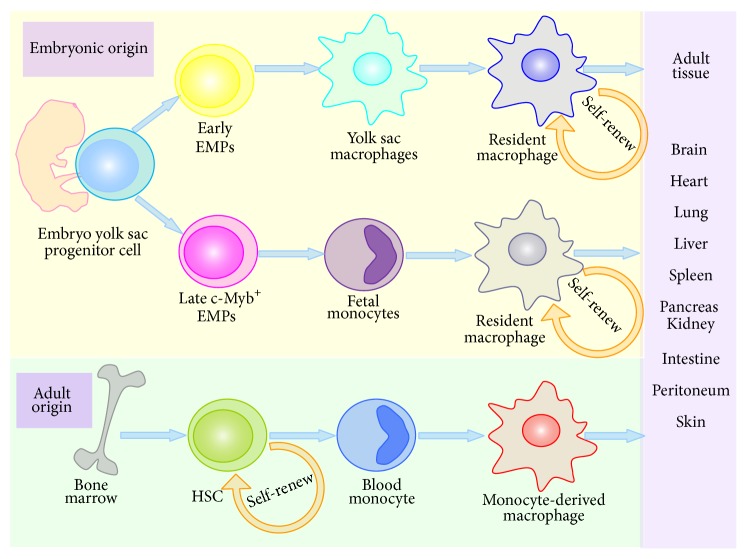
Origin and self-renewal of macrophage. Tissue macrophages have dual origins. One part develops from embryonic progenitors in the yolk sac and fetal liver and self-renew. The other part derives from hematopoietic stem cells (HSCs) in bone marrow and blood monocyte intermediates. HSCs also can self-replenish themselves. Monocyte-derived macrophages can give rise to some subsets of resident macrophages under certain conditions. Resident macrophages and monocyte-derived macrophages ultimately constitute macrophages in all tissues, such as microglia in the brain, Langerhans cells in the skin, and Kupffer cells in the liver. EMPs, erythromyeloid progenitors; HSCs, hematopoietic stem cells.

Historically, macrophages were considered to derive primarily from hematopoietic stem cells (HSCs) via bone marrow progenitors and circulating blood monocytes intermediates [4]. However, more and more evidences have revealed that there are dual origins of tissue macrophages, either from embryonic progenitors or from blood monocytes (Figure 1). The major macrophage populations are established prior to birth [5]. These cells develop from either primitive yolk sac macrophages or embryonic fetal liver monocytes and self-replenish themselves [1, 6]. Hoeffel and colleagues have shown that yolk sac macrophages derive from early erythromyeloid progenitors (EMPs), while late c-Myb^+^ EMPs seed the fetal liver and give rise to fetal monocytes. Both early EMPs and late c-Myb^+^ EMPs are generated in the yolk sac [6]. Yolk sac macrophages are the main precursors of microglia, while fetal monocytes differentiate into most other macrophages (alveolar macrophages in the lung and Kupffer cells in the liver, for example) [6–8]. In dermis and gut tissues, macrophages are renewed by adult HSC-derived monocytes [9, 10]. Besides, in spleen, kidney, and pancreas, macrophages with dual origins coexist [11]. However, most studies on origin of macrophages are focused on rodents and cells, so the exact origin of human macrophages is urgent to be clarified.

### 2.2. The Polarization and Roles of Macrophages

**Figure 2:**
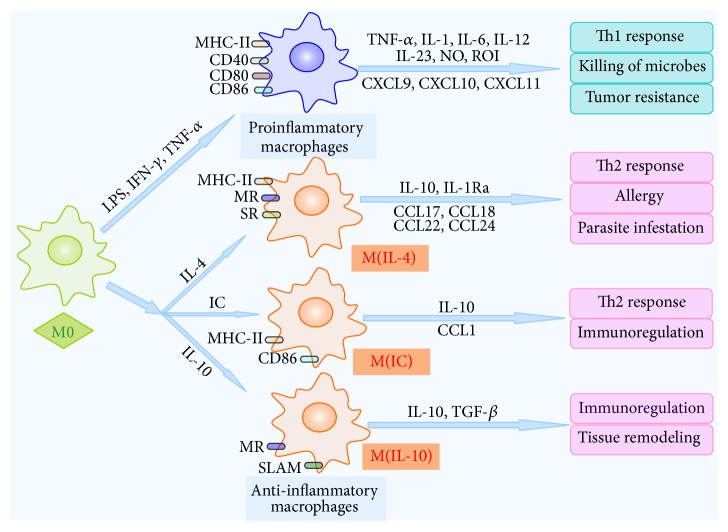
Macrophage polarization into proinflammatory and anti-inflammatory macrophages. Macrophages polarize and acquire different functional properties in response to numerous factors from the microenvironment. Macrophages activated by IFN-*γ*, LPS, or TNF-*α* can develop proinflammatory macrophages, with strong microbicidal and tumoricidal properties. In contrast, anti-inflammatory macrophages contribute to Th2 response, immunoregulation, and tissue remodeling. Anti-inflammatory macrophages have different subsets. M(IL-4) macrophages (induced by exposure to IL-4) secret TNF-*α*, IL-1, and IL-6 and induce Th2 cell response and allergy. M(IC) macrophages (induced by IC) secret IL-10 and exert immunoregulatory function. M(IL-10) macrophages (induced by IL-10) secret IL-10 and TGF-*β*, suppress immune responses, and promote tissue remodeling. CCL, CC-chemokine ligand; CXCL, CXC-chemokine ligand; IC, immune complexes; IFN-*γ*, interferon *γ*; LPS, lipopolysaccharide; MHC-II, major histocompatibility complex-II; MR, mannose receptor; NO, nitric oxide; ROI, reactive oxygen intermediates; SLAM, signaling lymphocytic activation molecule; SR, scavenger receptor; TGF-*β*, transforming growth factor-*β*; TLR, toll-like receptor; TNF-*α*, tumor necrosis receptor-*α*.

Macrophages not only present antigens as other antigen presenting cells (APCs) such as dendrite cells, but also eliminate microbes and tumor cells together with natural killer cells, T cells and B cells. What is more, macrophages contribute to tissue repair and remodeling, as well as restoration of pathogen-disturbed homeostasis [12]. The activated state, or polarization, of the macrophages depends on numerous factors from the microenvironment they reside in during normal homeostasis and in the pathological conditions [3]. Pathogen- and self-local environment-derived stimuli induce the macrophage phenotypic polarization [13]. Proinflammatory subtype/anti-inflammatory subtype polarization is the most well-described and commonly reported paradigm of macrophage polarization [14] (Figure 2). Proinflammatory subtype, also known as classically activated macrophages, is generally instigated by the presence of microbial products, such as lipopolysaccharide (LPS), proinflammatory cytokines, interferon-*γ* (IFN-*γ*), and tumor necrosis factor-*α* (TNF-*α*), as well as damage associated molecule patterns high mobility group box 1. Anti-inflammatory subtype, regarded as alternative activated macrophages, is activated by T helper 2 (Th2) cell-associated cytokines (IL-4 and IL-13), anti-inflammatory molecules (IL-10 and glucocorticoids), and immune complexes (IC) [15, 16]. Proinflammatory macrophages, characterized by their expression of high levels of TNF-*α*, IL-1, IL-6, IL-12, IL-23, nitric oxide (NO), and reactive oxygen intermediates (ROI), by their upregulation of major histocompatibility complex-II (MHC-II), costimulatory molecules, and T helper 1- (Th1-) recruiting chemokines, have a strong microbicidal and tumoricidal activity [17–19]. By contrast, anti-inflammatory macrophages, which upregulate surface molecules including mannose receptor CD206 and scavenger receptor CD163 and produce high levels of IL-10, transforming growth factor-*β* (TGF-*β*), and chemokines, are supposed to contribute to parasite infestation, tissue remodeling, and tumor progression [14, 17, 19, 20]. Anti-inflammatory macrophages can be further subcategorized into M(IL-4), M(IC), M(IL-10), and so on [15, 19]. M(IL-4), activated by IL-4, produces CCL24 and CCL22 in mice and CCL17 and CCL18 in human, resulting in the recruitment of eosinophils, basophils, and Th2 cells [19]. M(IC), stimulated by immune complexes (IC), produces CCL1 in mice, recruiting regulatory T cells (Tregs) [19]. M(IL-10) is activated by IL-10, which is immunosuppressive and engaged in extracellular matrix remodeling [14]. Diverse microenvironmental factors shape macrophage different activation states, which induce the dynamic switch of macrophage phenotype and function, showing different extremes of a continuum ranging from proinflammatory subtype to anti-inflammatory subtype [21, 22]. Transcription factors including STAT1, STAT6, C/EBPb, IRF-4, IRF-5, and PPAR-*γ* can regulate transcription programs which control the polarization of proinflammatory/anti-inflammatory macrophage [23, 24]. Proinflammatory subtype/anti-inflammatory subtype polarization status is regulated by the complex and interacting endogenous cellular signaling pathways in the microenvironment, such as C-Jun N-terminal kinase (JNK) signaling pathway, phosphatidylinositol-3-kinase (PI3K)/Akt signaling pathway, Notch signaling pathway, and JAK/STAT signaling pathway [2].

Macrophages are dispersed in many tissues and have distinct functions influenced by their location in the body [25]. Kupffer cells in liver contribute to the uptake of lipoprotein for maintenance of homeostasis and the endocytosis of pathogens and waste materials for host defense [26]. Alveolar macrophages in lung are involved in the uptake of inhaled particle and host defense against many borne microorganisms [27]. In homeostasis, Kupffer cells achieve immune surveillance and liver tolerance through IL-10 secretion [28]. Perturbation of homeostasis results in the activation of Kupffer cells by *β*-glucans from bacteria and fungi or lipopolysaccharide (LPS), the endotoxins of Gram-negative intestinal bacteria [29]. Activated Kupffer cells present either proinflammation or anti-inflammation phenotype [17]. Upon activation, microglia acquire an amoeboid shape and exert proinflammation or anti-inflammation roles dependent on different cytokines and other mediators they are exposed to [30].

In disease state, identifying different subsets of macrophages, activated states of macrophages, and macrophage polarization is crucial for understanding the pathogenesis and treatment of human disease.

## 3. Macrophages in Human Neuroimmune Diseases and Their Animal Models

Macrophages represent a ubiquitous yet complex population of immune cells that play major roles in both disease and homeostasis throughout the body. They contribute to both pathology and resolution in all acute and chronic inflammatory diseases including infections, cancer, obesity, atherosclerosis, and autoimmune disorders [31]. Neuroimmune diseases are a series of complex autoimmune diseases which involve the nervous system, including MS, NMO, GBS, and MG. The exact pathogenesis of these diseases is essentially ambiguous. But emerging data has suggested that macrophages may be associated with the development of these diseases (Table 1).

### 3.1. Macrophages in Multiple Sclerosis and Experimental Autoimmune Encephalomyelitis

MS, one of the most frequent central nervous system (CNS) diseases in young adults, is a progressive autoimmune disease caused by damage to the myelin and axons of brain and spinal cord [56]. MS patients show various neurological symptoms which originate in different areas of the CNS, such as motor deficits, sensory disturbances, visual disturbances, and neuropsychological symptoms [57]. So far, the etiology of MS is still not well understood; genetic, metabolic, environmental, and immunological factors have all been implicated [58]. The pathological hallmarks of MS consist of lymphocytes and macrophage infiltration, axonal demyelination, neuronal impairment, and remyelination [59, 60]. Different functional subpopulations of macrophages, with various roles including phagocytosis, antigen presentation, and lymphocyte stimulation, are abundantly present in inflammatory MS lesions [61]. Macrophages not only induce lesion formation and axonal damage, but also contribute to remyelination. On one hand, macrophages exert proinflammatory, neurotoxic, and myelin-attacking properties through secretion of inflammatory mediators, reactivation of pathogenic T cells, and suppression of Tregs expansion [32]. On the other hand, macrophages present repair mechanisms through the production of neurotrophic factors and clearance of myelin debris [33, 34]. Experimental autoimmune encephalomyelitis (EAE) is an animal model used to explore the mechanisms of MS and translate them into therapeutic interventions [62]. EAE can be induced either by active immunization with myelin components coupled with adjuvant or by passive transfer of myelin-reactive T cells [63]. EAE shares many pathological features with MS, such as chronic demyelination, neuronal damage, and neuroinflammation [64, 65]. It has been demonstrated that macrophages have a pathogenic role in initiating EAE, and eliminating macrophages significantly inhibits disease [35]. Another study showed that macrophages predominated in demyelinated areas and the macrophage number was correlated with tissue damage in EAE [36]. However, macrophages are also beneficial to remyelination. Undoubtedly, macrophages in MS or EAE consist of different phenotypical and functional subpopulations (Table 2).

#### 3.1.1. Microglia and Monocyte-Derived Macrophages

Historically it was difficult to distinguish activated microglia from activated macrophages in CNS lesion sites because they both present similar antigenic markers [87]. Thanks to chimeric mice, whose bone marrow (BM) cells are replaced by donor BM cells containing mismatched-MHC of fluorescently labeled myeloid cells, microglia can be distinguished from monocyte-derived macrophages [88, 89]. Microglia and monocyte-derived macrophages are functionally distinct populations of macrophages with unique origins. Microglia are located in the parenchyma and rely on local self-renewal, while monocyte-derived macrophages are renewed by blood derived monocytes and situated in both the parenchyma and the CNS barriers of the choroid plexus, perivascular space, and the meninges [30]. In addition, a TGF*β*-1 dependent microglial signature of microglia can provide the ability to distinguish microglia from infiltrating myeloid cells in the CNS [90]. Also, an evolutionarily conserved protein TMEM119 serves as a reliable microglial marker that differentiates microglia from monocyte-derived macrophages in human brain [91]. Interestingly, there is virtually no background trafficking of monocyte-derived macrophages in the CNS parenchyma of healthy organism [36]. Perturbation of CNS homeostasis can result in the recruitment of monocyte-derived macrophages which are associated with axonal loss, astrogliosis, and neurodegeneration in the CNS [30]. Once homeostasis is restored, these monocyte-derived macrophages seem to vanish [30]. A recent study revealed important physiological roles of microglia in learning and memory by promoting learning-associated synaptic structural remodeling using CX_3_CR1^CreER^ mice which express tamoxifen-inducible Cre recombinase [92]. Now it has been generally accepted that EAE is characterized by activation of resident microglia and extensive infiltration of monocyte-derived macrophages. Monocyte-derived macrophages are important in the effector phase of EAE and actively initiated demyelination. But the activation of microglia precedes the massive immune cells infiltration and the demyelination cascade and finally dominates the remyelination and repair of disease [93]. Microglia not only boost inflammatory and degenerative events in the CNS, which are correlated with axon and oligodendrocyte pathology, but also exert neuroprotective role in EAE [30]. Ponomarev et al. found that activated microglia promote the development and maintenance of inflammatory lesions in the CNS before the infiltration of circulating monocytes/macrophages into the CNS, implying the contributions of microglia in the early stages of EAE [78]. However, another study showed that microglia eliminated debris and suppressed cellular metabolism at EAE onset, presenting a beneficial role [36]. After myelin internalization, microglia gain a less-inflammatory phenotype and support tissue repair [94–96]. In addition, microglia express high levels of TGF-*β* and low levels of activation markers CD45, CCR1, and CCR5, which induces a protective process [37]. Monocyte-derived macrophages are phagocytic and inflammatory cells which initiate demyelination at EAE onset [36]. Monocyte-derived macrophages can present antigens and activate myelin-reactive T cells in CNS of EAE and then express high levels of adhesion molecules (ICAM-1 and VCAM-1) and chemokines (CCL2 and CCL3), attracting leukocyte infiltration into CNS [79–81]. Moreover, monocyte-derived macrophages induce the activation of resident microglia to accelerate inflammation, indicating that they are important population in EAE pathology [82]. These results show that macrophages play a key role in disease processes. The intervention of macrophage/microglia activation prior to disease induction had modest effects in disease progression; nevertheless the intervention at disease onset significantly improved disease severity [97]. Furthermore, inhibiting the activation of microglia induced a delayed onset of EAE [98]. Another study showed that conditional depletion of microglia-endogenous TGF-*β*-activated kinase 1 (TAK1) suppressed disease, strongly diminished CNS inflammation, and decreased tissue damage by cell-autonomous inhibition of the NF-*κ*B, JNK, and ERK1/2 pathways in EAE [99, 100]. Through CD11b-HSVTK mice which express herpes simplex thymidine kinase in macrophages and microglia, Heppner et al. found that microglial paralysis suppressed the development and maintenance of inflammatory CNS lesions in EAE [101]. A recent study has demonstrated that CXCR7 suppression modulated microglial chemotaxis to ameliorate the clinical severity of EAE [102]. In addition, hydroxychloroquine treatment suppressed the activation of human microglia and attenuated EAE [103]. 18*β*-glycyrrhetinic acid can attenuate EAE through suppressing microglia activation-mediated CNS inflammation and promoting neuroprotective roles of microglia [104]. Fingolimod treatment of EAE resulted in diminished microglial activation in vivo PET imaging [105]. From these studies, it has been speculated that microglia/macrophages, which display double roles in the disease course of EAE, are quite important for exploring the pathogenesis and progression of MS.

In MS, microglia turn into competent APCs for T cells after eating myelin and axonal remnants, which promote their expression of MHC-II and costimulatory molecules and their secretion of inflammatory and neurotoxic molecules, resulting in neuroinflammation and demyelination [66, 67]. Moreover, microglia play a crucial role in the maintenance of CNS homeostasis [68]. Microglia in normal appearing white matter of MS patients display features of immunosuppression and expressed molecules to prevent activation and tissue damage [69]. Monocyte-derived macrophages are found in active demyelinating lesions of MS patients [106, 107]; one part contains myelin remnants [70] and the other secretes inflammatory cytokines and expressed costimulatory molecules, both inducing MS lesion development [71, 72]. Besides, some monocyte-derived macrophages display an intermediate activation which suppress neuroinflammation and promote CNS repair, presenting a neuroprotective role in MS [73, 74]. Glucocorticoids, IFN-*β*, glatiramer acetate, and fingolimod, commonly used drugs for MS, can effectively inhibit macrophage or microglia activation and alleviate disease severity in early stage of MS [108–111]. Therefore, targeting macrophages or microglia is an attractive therapeutic option for the treatment of MS.

#### 3.1.2. Proinflammatory and Anti-Inflammatory Microglia/Macrophages

The current concept of macrophage polarization describes two subtypes with distinct but opposing functions [112], the proinflammatory subtype with secretion of TNF-*α*, IL-1*β*, IL-12, and IL-23 and the anti-inflammatory subtype with secretion of IL-10, TGF-*β*, and sIL-1R*α* [113–115]. It has been demonstrated that proinflammatory microglia/macrophages induce tissue damage due to excessive secretion of proinflammatory cytokines, ROI and NO [75, 76]. In contrast, anti-inflammatory microglia/macrophages can phagocytose debris and promote tissue repair and termination of neuroinflammation, leading to a neuroprotective response [77].

In EAE, microglia/macrophages also can be classified into proinflammatory and anti-inflammatory microglia/macrophages. Proinflammatory and anti-inflammatory microglia/macrophages predominate differentially during disease course. For instance, proinflammatory microglia/macrophages contribute to the establishment of early inflammation in EAE, whilst anti-inflammatory microglia/macrophages induce the resolution of inflammation [83]. What is more, proinflammatory microglia/macrophages are associated with increased EAE severity, whereas anti-inflammatory microglia/macrophages are correlated with ameliorated clinical disease [84]. Anti-inflammatory microglia/macrophages promote the differentiation of Th2 cells and Tregs, which can suppress EAE severity [85]. Anti-inflammatory microglia/macrophages also participate in the development of relapses in EAE [116]. Administration of ex vivo activated anti-inflammatory macrophages may not only suppress ongoing severe disease but also promote immunomodulatory expression pattern in CNS lesions, indicating their anti-inflammatory role in the recovery of EAE [116]. Adoptive transfer of anti-inflammatory macrophages could inhibit the development of T helper 17 (Th17) cells and induce the differentiation of Th2 cells and Tregs which both reverse EAE, confirming their direct therapeutic relevance [85, 86].

Recent studies also have shown that there are CD163^+^ and Arg-1^+^ anti-inflammatory microglia/macrophages in MS brain [94, 117]. In addition, primary cultures of human monocyte-derived macrophages were exposed to IFN-*γ* and LPS for the activation of M1 and to IL-4 for the activation of anti-inflammatory macrophages. Anti-inflammatory macrophages migrated over longer distance and with higher velocity towards CCL5, CXCL10, CXCL12, and C1q, all of which were key factors for monocytes recruitment into MS lesions, whereas proinflammatory macrophages did not respond and remained sessile [118]. Upon stimulation with CCL2, anti-inflammatory macrophages were able to make filopodia, while proinflammatory macrophages adapted a spherical morphology, suggesting that the cytoskeleton of proinflammatory and anti-inflammatory macrophages was rearranged [118]. So, the activation status of macrophage induced the cytoskeleton rearrangement and affected macrophage migration, which may involve the pathological process of MS [118]. Intriguingly, another study showed that, in active demyelinating MS lesions, although macrophages and activated microglia predominantly displayed proinflammatory characteristics, the majority of these cells coexpressed the markers of proinflammatory and anti-inflammatory macrophages, suggesting an intermediate activation status [59]. The balance between proinflammatory and anti-inflammatory microglia/macrophages is proposed to predict the development of disease and relapse [66]. Furthermore, anti-inflammatory microglia/macrophages are increased in MS after treatment with glatiramer acetate. Induction of anti-inflammatory microglia/macrophages may suppress neuroinflammation and promote CNS repair. Hence, the treatment of MS may focus on shifting proinflammatory microglia/macrophages into anti-inflammatory microglia/macrophages.

In conclusion, the double roles of microphages and identifying the beneficial subset in disease course should be clarified. Of course, future studies should shed light on the double roles of microglia and CNS-infiltrating macrophages, proinflammatory and anti-inflammatory microglia/macrophages in different stages of disease process, and the cell intrinsic and extrinsic pathways that regulate the roles and phenotype change. Most of all, shifting the phenotype of macrophages into the beneficial one is an attracting therapeutic hint.

### 3.2. Macrophages in Neuromyelitis Optica and Its Animal Model

NMO is a neuroimmune disorder characterized by recurrent episodes of optic neuritis and transverse myelitis, resulting in significant blindness and/or paralysis [119]. Antibodies against aquaporin-4 (AQP4) are found in the serum of most NMO patients [120]. AQP4 is a water channel protein expressed on astrocytic end-feet in CNS, as well as skeletal muscle cells and epithelial cells in kidney, lung, and gastrointestinal tract [121]. Anti-AQP4 autoantibody (NMO-IgG) plays a key role in the pathogenesis of NMO [122]. NMO-IgG binds to AQP4 on astrocytes, then induces complement-dependent cytotoxicity (CDC) and antibody-dependent cellular cytotoxicity (ADCC), and finally leads to blood-brain barrier disruption, demyelination, and neuronal injury [38]. The pathological features of NMO include vasculocentric deposition of immunoglobulin and activated complement, loss of AQP4 and glial fibrillary acidic protein, marked granulocyte and macrophage infiltration, and demyelination with axon loss [39]. Macrophages also participate in CDC and ADCC of NMO. So far, no single rodent model has proven to be a perfect representation of NMO in humans [123]. Commonly used experiments are obtained through passive transfer of NMO-IgG in certain contexts in rats or spinal cord cultures [124]. A study showed that macrophages exacerbated the severity of NMO lesions in spinal cord cultures exposed to NMO-IgG and complement [41]. In a model of NMO in rats produced by intracerebral injection of NMO-IgG, depletion of monocytes and macrophages (both proinflammatory and anti-inflammatory subtypes) could reduce the severity of NMO pathology [42]. Macrophages exacerbate astrocyte damage of NMO lesions through phagocytosis and secretion of proinflammatory cytokines or oxidative metabolites [33]. In the brain lesions of patients with NMO, CD68^+^ macrophages and microglia expressed intense immunoreactivities for interferon gamma-inducible protein 30 (IFI30) and CD163, suggesting that severe fulminant activation of macrophage-mediated proinflammatory immune mechanism exerted a crucial role in the generation of NMO lesions [40].

Only a few of studies have shown that macrophages involve NMO and its animal models, let alone the roles of different subsets of macrophages, such as microglia/macrophages, and the different polarization of macrophages in NMO. Future studies should focus on the roles of macrophage subsets and clarify whether macrophages can become the therapeutic target of NMO.

### 3.3. Macrophages in Myasthenia Gravis and Experimental Autoimmune Myasthenia Gravis

MG, an antibody-mediated neuroimmune disease of the neuromuscular junction, is characterized by fluctuating muscle weakness and abnormal fatigability [125]. Pathogenic autoantibodies consist of antibodies against acetylcholine receptor (AChR), muscle-specific tyrosine kinase (MuSK), lipoprotein receptor-related protein 4 (LRP4), and so on [126]. The autoantibodies are produced in T cell dependent and B cell mediated pathogenic processes, which further activate the complement system and induce inflammation of the postsynaptic muscle membrane. The abnormalities of the thymus are related to the pathogenesis of MG, including thymoma and thymic hyperplasia [127]. Experimental autoimmune myasthenia gravis (EAMG), induced by immunization with Torpedo AChR, is a conventional animal model of MG, commonly used to investigate the mechanism underlying the pathophysiology of MG for the development of novel therapeutic strategies [128]. A previous study indicated that the pathologic features of EAMG in the acute phase included macrophage infiltration and inflammation of muscle endplates and muscle fiber necrosis [44]. Macrophages act as APCs during the acute phase of EAMG, while they promote the production of antibodies to self-AChR in the chronic phase [45]. However, large suppressive macrophages generated from restimulating spleen cells from EAMG could induce apoptosis in activated T cell blasts in vitro, indicating a potential immunotherapy of EAMG [46]. In human, there are poliovirus-infected macrophages in thymus of several MG patients, which may be involved in the intrathymic alterations leading to MG [43]. Future studies may be conducted with respect to analysis of the macrophage subsets and polarization in the pathogenesis and treatment of MG.

### 3.4. Macrophages in Guillain-Barré Syndrome and Experimental Autoimmune Neuritis

GBS is an acute inflammatory demyelinating neuropathy, resulting from a complicated immune response to incompletely characterized antigens in the peripheral nervous system [129]. Acute inflammatory demyelinating polyneuropathy (AIDP) and acute motor axonal neuropathy (AMAN) are typical subsets of GBS [47]. Both cellular and humoral immunity contribute to disease development, resulting in neuroinflammation, demyelination, and axonal damage in the peripheral nervous system (PNS) [47, 130]. AIDP is majorly related to CD4^+^ T cell induced macrophage associated demyelination, while AMAN mostly involves autoantibodies against ganglioside [48]. Experimental autoimmune neuritis (EAN) which is a T cell mediated inflammatory demyelinating disease induced by immunization with proteins and peptides of PNS myelin together with Freund's complete adjuvant is regarded as a useful animal model of GBS [131, 132].

Macrophages exercise their functions through professional antigen presentation and secretion of cytokines and other inflammatory mediators [47, 49, 50]. Macrophages express high levels of MHC-II in EAN [131]. What is more, macrophages secrete proinflammatory cytokines IL-12 and TNF-*α*, matrix metalloproteinase-9 (MMP-9), and inducible nitric oxide synthase (iNOS), which propagate inflammation and induce myelin and axonal damage in EAN [52, 53]. Interestingly, macrophages in PNS not only contribute to the inflammatory pathology and tissue destruction, but also promote recovery in EAN [52]. In EAN, macrophages induce T cell apoptosis by secreting proapoptotic mediators if they contact with their targets [54]. Macrophages also secrete IL-10 and TGF-*β* in EAN, which both inhibit the disease and reduce disease severity [48, 55]. What is more, macrophages are involved in the pathogenesis of GBS. Macrophages phagocytose myelin in AIDP and axons in AMAN [51]. Macrophage-mediated segmental demyelination and axonal loss are the pathological features of GBS [133]. Macrophages express high levels MHC-I and MHC-II in GBS [134]. In addition, macrophages are directed towards myelin or axonal targets by antibodies and attack targets in a complement-dependent manner [53]. Interestingly, macrophages in PNS promote recovery in GBS [48].

There are resident endoneurial and monocyte-derived macrophages in GBS and EAN. Different from microglia, most of these resident macrophages in the PNS are renewed by monocyte-derived macrophages [135]. In PNS, resident endoneurial macrophages express MHC-I, MHC-II, and complement receptors [136]. Monocyte-derived macrophages are important for full-brown inflammatory disease in EAN because elimination of these cells reduced disease severity [137]. A study indicated that TNF-*α* exacerbated EAN by inducing proinflammatory macrophages. However, TNF-*α* deficiency attenuated EAN by inducing a switch of macrophage phenotype from proinflammatory subtype to anti-inflammatory subtype [52]. Similarly, compound A which is a plant origin ligand of glucocorticoid receptors also could relieve the severity of EAN by inducing anti-inflammatory macrophages [138].

So, it is better to understand the roles of resident and blood derived macrophages, as well as M1 and M2 cells in the development of GBS and EAN.

## 4. Conclusion

Macrophages, both proinflammatory and anti-inflammatory, participate in the complex immunopathological framework in the pathologies of neuroimmune diseases. The change of microenvironment in disease process dictates macrophage polarization, such as functional and hypotypic differentiation. Future studies are needed for the exploration of the exact double roles of macrophage subsets and the shift between them, indicating a macrophage-centered therapeutic strategy for neuroimmune disorders.

## Figures and Tables

**Table 1:** Roles of macrophages in neuroimmune diseases and their animal models.

Neuroimmune diseases	Beneficial roles of macrophages	Harmful roles of macrophages	Refs
MS	Production of neurotrophic factors Clearance of myelin debris	Secretion of inflammatory mediators Reactivation of pathogenic T cells Suppression of Tregs expansion	[32–34]

EAE	Eliminated debris and suppress cellular metabolism Production of anti-inflammatory TGF-*β*	Presenting a pathogenic role in initiating EAE Eliminating macrophages inhibits EAE A positive correlation between macrophage number and tissue damage in EAE	[35–37]

NMO		Participate in CDC and ADCC Mediating proinflammatory immune mechanism through the expression of intense immunoreactivities for IFI30 and CD163	[38–40]

Rats or spinal cord cultures models for NMO		Macrophages exacerbated the severity of NMO lesions in spinal cord cultures Depletion of macrophages reduced the severity of NMO pathology in rats Phagocytosis Secretion of proinflammatory cytokines	[33, 41, 42]

MG		Poliovirus-infected macrophages in thymus of several MG patients, which may be involved in the intrathymic alterations leading to MG	[43]

EAMG	Induction of apoptosis in activated T cell blasts in vitro by large suppressive macrophages which were generated from restimulating spleen cells from EAMG	Acting as APCs during the acute phase Promoting the production of antibodies to AChR in the chronic phase	[44–46]

GBS	Secretion of anti-inflammatory cytokine IL-10	Professional antigen presentation Secretion of cytokines and other inflammatory mediators Phagocytosing myelin in AIDP and axons in AMAN	[47–51]

EAN	Inducing T cell apoptosis by secreting proapoptotic mediators Secretion of IL-10 and TGF-*β* which both can reduce the severity of EAN	Acting as APCs Secreting proinflammatory cytokines IL-12, TNF-*α*, MMP-9, and iNOS that propagate inflammation and induce myelin and axonal damage Attacking myelin or axon in a complement-dependent manner	[52–55]


AChR, acetylcholine receptor; ADCC, antibody-dependent cellular cytotoxicity; AIDP, acute inflammatory demyelinating polyneuropathy; AMAN, acute motor axonal neuropathy; APCs, antigen presenting cells; CDC, complement-dependent cytotoxicity; EAE, experimental autoimmune encephalomyelitis; EAMG, experimental autoimmune myasthenia gravis; EAN, experimental autoimmune neuritis; GBS, Guillain-Barré syndrome; IFI30, interferon gamma-inducible protein 30; IL-10, interleukin-10; IL-12, interleukin-12; iNOS, inducible nitric oxide synthase; MMP-9, matrix metalloproteinase-9; MS, multiple sclerosis; NMO, neuromyelitis optica; TGF-*β*, transforming growth factor-*β*; TNF-*α*, tumor necrosis receptor-*α*; Tregs, regulatory T cells.

**Table 2:** Roles of different functional subpopulations of macrophages in MS and EAE.

	Tissue resident macrophages (microglia)	Monocyte-derived macrophages	Proinflammatory macrophages	Anti-inflammatory macrophages	Refs
MS	As competent APCs Secretion of proinflammatory and neurotoxic molecules Maintenance of CNS homeostasis Immunosuppression	Eating myelin remnants Secreting proinflammatory cytokines Displaying an intermediate activation to suppress neuroinflammation and promote CNS repair	Excessive secretion of proinflammatory cytokines, ROI and NO	Phagocytosing debris Promoting tissue repair Increased anti-inflammatory macrophages in MS after treatment with glatiramer acetate	[66–77]

EAE	Promoting the development and inflammatory lesions in CNS in the early stage of EAE Eliminating debris and suppressing cellular metabolism at EAE onset Secretion of TGF-*β* to induce a protective process	Presenting antigen Activating myelin-reactive T cells Expressing adhesion molecules and chemokines to attract leukocyte infiltration into CNS Activating some microglia to accelerate inflammation	Contributing to the establishment of early inflammation in EAE Associating with EAE severity	Promoting the differentiation of Th2 cells and Tregs to suppress EAE severity Inhibiting the development of Th17 cells	[30, 36, 37, 78–86]

APCs, antigen presenting cells; CNS, central nervous system; EAE, experimental autoimmune encephalomyelitis; MS, multiple sclerosis; NO, nitric oxide; ROI, reactive oxygen intermediates; TGF-*β*, transforming growth factor-*β*; Th2, T helper 2; Th17, T helper 17; Tregs, regulatory T cells.
